# Prevalence and Behavioral Factors Associated with Penile and Rectal HPV Infection among Men Who Have Sex with Men in Kenya

**DOI:** 10.21203/rs.3.rs-9335695/v1

**Published:** 2026-04-23

**Authors:** Kate Klein, Fredrick O. Otieno, Walter Agingu, Patriciah Wambua, Felix Ochieng, Minjee Lee, Lyle R. McKinnon, Lifang Hou, Supriya D. Mehta

**Affiliations:** 1 Havey Institute for Global Health, Northwestern University, Chicago, USA; 2 Nyanza Reproductive Health Society, Kisumu, Kenya; 3 Department of Medical Humanities and Social Medicine, Kosin University College of Medicine, Busan, South Korea; 4 Departments of Medical Microbiology and Infectious Diseases, University of Manitoba, Winnipeg, Canada; Centre for the AIDS Programme of Research in South Africa (CAPRISA), Kwazulu-Natal, South Africa; Department of Medical Microbiology and Immunology, University of Nairobi, Nairobi, Kenya; 5 Department of Internal Medicine, Rush University, Chicago, USA

**Keywords:** High-risk human papillomavirus, Men who have sex with men, Rectal infection, Penile infection, Bacterial sexually transmitted infections, HIV co-infection, Kenya, Sub-Saharan Africa

## Abstract

**Background:**

High-risk human papillomavirus (hrHPV) is a leading cause of anogenital cancers and disproportionately affects men who have sex with men (MSM), particularly in low- and middle-income countries. Few studies in sub-Saharan Africa have concurrently examined penile and rectal hrHPV infection and their site-specific behavioral and biological correlates. We assessed the prevalence, genotype distribution, and factors associated with penile and rectal hrHPV among MSM in Kisumu, Kenya.

**Methods:**

We conducted a cross-sectional analysis of baseline data from MSM aged 18–35 years. Clinician-collected penile and rectal swabs were tested for hrHPV using the ScreenFire HPV assay. Urine and rectal specimens were tested for *Chlamydia trachomatis* and *Neisseria gonorrhoeae* using GeneXpert, and HIV status was confirmed as per Kenyan national guidelines. hrHPV was analyzed separately by anatomical site. Log-binomial regression was used to estimate adjusted prevalence ratios (aPR) for associations with sociodemographic, behavioral, and biological factors.

**Results:**

Penile hrHPV prevalence was 24.7% (39/158) and rectal hrHPV prevalence was 12.5% (22/176); 6.7% of participants tested at both sites had dual-site infection. Most infections at both sites involved a single genotype group. HPV-18/45 was the most common genotype group at both sites. Penile hrHPV was more likely among participants with HIV (aPR=2.27, p=0.050) and those reporting insertive anal sex (aPR=5.26, p=0.062), and was less common among employed participants (aPR=0.25, p=0.048). Circumcision showed a protective trend (aPR=0.52, p=0.090). Rectal hrHPV was more common among participants with rectal chlamydia and/or gonorrhea infection (aPR=3.34, p=0.001), transgender or non-binary identity (aPR=2.28, p=0.036), and receptive anal intercourse (aPR=3.97, p=0.062). Although HIV was associated with rectal hrHPV in unadjusted analyses, this relationship was attenuated after accounting for rectal bacterial STI infection.

**Conclusions:**

hrHPV infection was common among MSM in Kisumu, Kenya, with distinct site-specific correlates. The strong association between rectal bacterial STIs and rectal hrHPV highlights the need for integrated HPV and STI screening. These findings support expansion of gender-neutral HPV vaccination and etiologic STI screening to reduce HPV-associated cancer risk among MSM in sub-Saharan Africa.

## Introduction

High-risk human papillomavirus (hrHPV) infection of the anal and penile epithelium is a significant global public health concern, particularly among men who have sex with men (MSM), including those living with HIV, and those residing in low-and middle-income countries (LMICs). HPV infects epithelial cells of mucosal and cutaneous tissues, including the cervix, anus, penis, and oropharynx. Persistent infection with hrHPV strains, notably HPV-16 and HPV-18, can lead to cancerous cell development. In LMICs, where screening for hrHPV is limited, mortality from cervical, anal and oropharyngeal cancers remains disproportionately high (WHO, 2024). Relatively few studies examine both anal and penile HPV among MSM or the associated behavioral factors. The majority of these studies take place in countries outside of sub-Saharan Africa. Pooled analyses of 21,018 HIV positive and negative MSM demonstrate that anal hrHPV infection is common, with prevalence rates of 74.3% for HIV-positive MSM compared with 41.2% for HIV-negative MSM (Wei et al., 2021). We could not find a pooled prevalence estimate of penile hrHPV, but one study of 639 HIV-negative MSM found a prevalence of one or more hrHPV types was 17.9% (Nadar, et al. 2021). A meta-analysis of 2012–2019 data among MSM found the most prevalent high-risk HPV types identified in the anus were HPV-16 (19.9%), HPV-51 (10.5%), HPV-18 (10.4%), HPV-52 (8.9%) and HPV-58 (8.8%). The most frequent HPV high-risk types in the penis were HPV-16 (4.9%), HPV-18 (3.2%), HPV-58 (2.8%), HPV-51 (2.4%) and HPV-52 (2.3%) (Farahmand et al., 2021). A study of anal and penile HPV testing among 465 HIV positive and negative MSM in China found that anal HPV (68%) was more common than penile HPV (37.8%). Factors associated with penile HPV included insertive anal sex and syphilis, while inconsistent condom use during receptive anal sex and HIV seropositivity were associated with anal HPV (Qian et al, 2017). A more recent study of incidence, persistence, and clearance of any anal and penile HPV amongst MSM in Taiwan found high incidence rates of anal and penile HPV infection, at (43.6 and 26.8 infections per 1,000 person-months, respectively.) Inconsistent condom use for receptive anal sex was associated with increased risk of incident anal HPV, while older age was associated with greater penile HPV incidence, and MSM reporting having multiple sex partners for receptive anal sex were less likely to clear anal HPV infection (Zhou, et al., 2023).

In sub-Saharan Africa, studies consistently demonstrate high prevalences of anal hrHPV among MSM, with genotype distributions that often differ from those reported outside the region. In Nigeria, among circumcised MSM, prevalence of any HPV was 72.7% with 48% positive for hrHPV (Nampota-Nkomba, et al., 2025). In South Africa, anal HPV was detected in 72.8% of MSM overall, including 57.6% prevalence of hrHPV, with HPV-16 as the most common oncogenic type and frequent multiple type infections; HIV-positive MSM had particularly high burdens (81.2% hrHPV among those with HIV) compared with HIV-negative MSM (Müller et al,. 2016). In a study of MSM in the Central African Republic, 69.1% had anal HPV, of whom 82.7% carried hrHPV. Of the hrHPV strains, HPV-35, HPV-58, HPV-59, and HPV-31 predominated, while HPV-16 and HPV-18 were relatively less common (Mboumba Bouassa et al. 2018). Data from Bamako, Mali reflect similar patterns, with 70.0% overall anal HPV prevalence and 80.0% of positive specimens containing at least one hrHPV type, with an average of 3.7 hrHPV genotypes per sample (Diallo et al. 2021). A cohort in Benin reported that 36.3% of HIV-negative MSM were positive for anal hrHPV (genotypes including HPV-16, 18, 31, 35, 39, 45, 51, 52, 56, 58, 59, 66, and 68), and multiple high-risk infections occurred in a minority, although specific genotype frequencies were not detailed (Agbonon et al. 2024). In Rwanda, penile hrHPV prevalence (35.0%) was reported alongside anal hrHPV (20.1%), with higher penile hrHPV among MSM living with HIV (55.2%) versus HIV-negative MSM (29.7%), with variation by genotype including HPV-33, HPV-39, HPV-45, HPV-56, and HPV-51 across sites (Murenzi et al. 2024). Across these settings and studies, HPV-16, HPV-35, HPV-58, and HPV-59 appear frequently among oncogenic types, and HIV infection, receptive anal intercourse, and multiple sexual partners are consistently associated with greater hrHPV prevalence (Agbonon et al. 2024; Mboumba Bouassa et al. 2018; Müller et al. 2016; Yaya et al., 2021).

MSM have concomitantly high rates of other sexually transmitted infections (STIs), including urethral and rectal chlamydia and gonorrhea. Studies among MSM in coastal Kenya show that most laboratory-confirmed rectal gonorrhea and chlamydia infections occur in individuals reporting no symptoms, and this is biologically plausible because rectal and pharyngeal infections often produce minimal inflammation and nonspecific symptoms that are easily unnoticed, meaning syndromic management would miss a large proportion of infections (Mofolorunsho et al., 2024; Sanders et al., 2010; Williams, et al., 2023). In the *Anza Mapema* cohort in Kisumu, incident urethral and rectal chlamydia and gonorrhea infections were common (18 cases per 100 person-years), and most infections detected by nucleic acid amplification testing were asymptomatic (Otieno et al. 2020). A recent systematic review and meta-analysis of studies from sub-Saharan Africa, including Kenyan cohorts, estimated a pooled prevalence of chlamydia and gonorrhea infections of approximately 27% among MSM, confirming a persistently high regional burden (Mofolorunsho et al. 2024). Data from women indicates that the inflammatory nature of these STIs contribute to hrHPV acquisition risk and persistence (Silins et al, 2005). Despite the high prevalence of STIs among MSM, few studies have examined the intersection of penile and anal hrHPV and STIs in this population. In a study of 1,352 HIV-infected MSM in Italy, past history of gonorrhea was associate with nearly two-fold increased odds of anal hrHPV positivity, but STI testing was not conducted (Bruzzesi et al, 2021). In a study of 2,705 men who have sex with women, aged 17–28, in Kisumu, Kenya, hrHPV was detected in 31.2% of penile glans samples, with HPV-16 being the most common type; laboratory detected chlamydia and gonorrhea were associated with nearly 2-fold increased odds of hrHPV detection in analyses adjusted for sociodemographics and sexual practices (Smith et al, 2010). We are unable to find similar studies among African MSM, though studies in Europe find high rates of hrHPV and laboratory detected STI co-infection (Silins et al., 2005; Ceccarani et al., 2024; Nugent et al., 2021).

This study adds to the smaller pool of research that explores the prevalence and genotype distribution of hrHPV by anatomical site and associated behavioral practices and laboratory-detected STIs among MSM in Sub Saharan Africa. Addressing this gap, the present study measured hrHPV infection at two anatomical sites (anal and penile) among MSM in Kenya, co-infection with STIs and HIV, and the associated sociodemographics and behavioral practices.

## Methods

### Ethical Statement

This study was approved by the institutional review boards of Jaramogi Odinga Oginga Teaching and Referral Hospital (JOOTRH, ISERC/JOOTRH/754/23), Rush University Medical Center (RUMC, #23060502-IRB1), University of Manitoba (UM, HS26665), and non-human subjects determination from Southern Illinois University (041540) and Northwestern (STU00224946) for having no human subjects contact and only receiving de-identified data. Written informed consent was obtained for all participants in their preferred language (English, DhoLuo, Kiswahili).

### Eligibility

Data for this analysis were derived from men enrolled in the *Mbili Pamoja* study. The *Mbili Pamoja* study enrolled 250 men in Kisumu between June 7 through October 15, 2024. Details of the study design have been published (Ochieng et al., 2026). Briefly, to be eligible for *Mbili Pamoja*, participants had to be assigned male at birth, aged 18–35 years and reported having anal sex with another man in the past 3 months. Because *Mbili Pamoja* was designed to study the penile and rectal microbiome, men with recent circumcision (within 6 months), or recent vomiting or diarrhea (within 2 weeks) were temporarily ineligible. After symptoms resolved, participants could come for rescreening while enrolment remained open.

### Data Collection

Data were collected on sociodemographic characteristics, sexual practices, and psychosocial characteristics at baseline and at 6- and 12- months post-enrollment, in the participant’s preferred language (English, DhoLuo, or Kiswahili).

### HPV Testing

hrHPV testing was conducted only on samples collected at baseline and thus only baseline data are used for this analysis. Clinicians collected the penile and rectal swabs using flocculated swabs, pre-moistened with sterile saline. hrHPV testing was conducted via the Atila BioSystems portable iAMP-PS96 and its validated ScreenFire Risk Stratification HPV Assay (Atila BioSystems, 2025; Hou et al., 2024; Zhang et al., 2020; Wang et al., 2024). The ScreenFire Assay detects hrHPV types, reported as: HPV-16; HPV-18/45 (composite); HPV31/33/35/52/58 (composite); or HPV39/51/56/59/68 (composite) (Atila Biosystems, 2025; Hernandez et al, 2024; Wang et al., 2025). 402 samples from Kisumu were collected and tested for penile (n=202) and rectal (n=200) hrHPV as a convenience sample of consecutively available participants based on availability of test supplies. Overall, 24 (12%) rectal sample results and 44 (22%) penile sample results were “Invalid”, with the majority of the invalid results occurring in the first month of testing, as the lab staff gained expertise in operating the machine.

### STI and HIV Testing

Urine specimens and clinician-collected rectal swabs were tested for *Chlamydia trachomatis* (CT) and *Neisseria gonorrhoeae* (NG) using GeneXpert (Cepheid, Sunnydale, California, US). HIV status was via self-report, and rapid confirmatory testing for those reporting themselves HIV-negative following Kenyan guidelines (Kenya Ministry of Health, 2021). All of these tests were conducted at the study site in Kisumu.

### Statistical Analysis

The distribution of penile and rectal hrHPV types is presented as frequency and percentage distributions, with intersections of type-specific hrHPV depicted by upset plot. All analyses were stratified by anatomic site of infection, i.e., penile vs. rectal. We sought to identify sociodemographic and behavioral practices associated with hrHPV infection. Variables were selected based on review of the literature and those with plausible association with hrHPV, in particular increased sexual exposure and co-infections. Sociodemographic factors included age, gender identity, educational attainment, and income. Biological factors included circumcision status, HIV status, and STI infection. Sexual practices included number of sex partners, condom use, group sex, transactional sex, and usual sexual position for male sex (receptive, insertive, versatile). We explored cleaning of anus before and after sex in relation to rectal HPV, as this may relate to rectal mucosal inflammation (Broedlow et al., 2026) or risk of STI (Hassan et al., 2018). For the number of sexual partners, we examined the total number of male partners, female partners, and all partners combined. For transactional sex, giving money or gifts for sex and receiving money or gifts for sex were analyzed as a composite due to high correlation of the two variables. Condom use was analyzed as use at last sexual interaction, and the number of times condoms were used with female or male partners.

To identify factors associated with infection, hrHPV status was dichotomized as positive vs. negative. Chi square tests were conducted for categorical and ordinal variables, t-Tests for continuous normally distributed variables, and rank sum tests for non-normally distributed continuous variables. Fisher’s exact test was used for any categorical variable with a cell size of 5 or less. Variables with a univariable p-value <0.20 were retained for consideration in the multivariable model, consistent with Hosmer and Lemeshow’s (2000) purposeful selection strategy, which recommends a more liberal threshold to avoid excluding potential confounders. A log binomial model was used to calculate the prevalence ratio PR of being hrHPV positive. Data were analyzed using Stata/SE 19 for Windows (Stata Corporation, College Station, Texas).

## Results

### Characteristics of Study Sample

Among participants with valid hrHPV test results, 158 men were included in the penile hrHPV analysis and 176 in the rectal hrHPV analysis. Most participants were aged 18–29 years, identified as cisgender men, and had completed at least secondary education. The majority were circumcised (90.5%). Most (65.3%) participants reported usually insertive positioning during sex. Overall, 6.5% of participants were HIV positive, with 13.1% having rectal or urethral chlamydia or gonorrhea infection (9.1% urethral STI, 3.4% rectal STI, 1.5% urethral and rectal STI). Having an HPV test was not associated with gender, educational attainment, sexual practices, HIV status or STI status, but participants with HPV testing were on average 1.9 years younger (Supplemental Table 1).

### Distribution of hrHPV types in Penis and Rectal Samples

**Figure 1.**
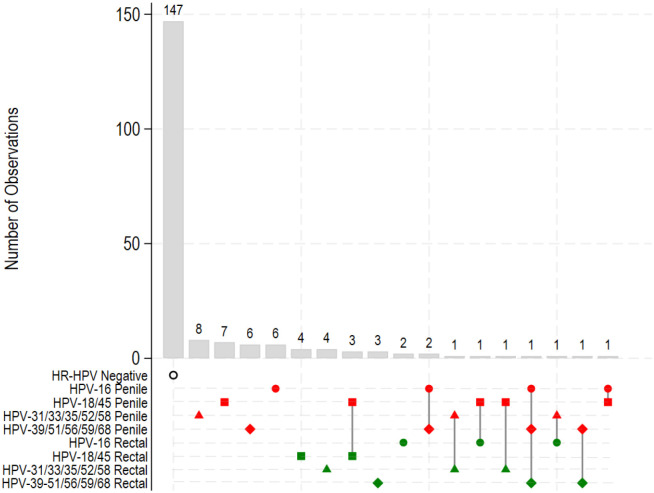
Distribution of hrHPV types in Penile and Rectal Samples Figure 1 shows the intersections of different penile and rectal hrHPV, with the rows corresponding to the status (e.g., HPV-16) labeled by anatomic site (penile [red] or rectal [green]), and the columns to the counts and intersections of these. The number of participants observed in the intersection is shown in the vertical bar with the number of participants labelled (y-axis and each bar). For example, there are n=6 participants with penile HPV-16 alone; n=2 with HPV-16 and HPV-39/51/56/59/58; n=1 with penile HPV-16, penile HPV-39/51/56/59/58 and rectal HPV-31/33/35/52/58; and n=1 with penile HPV-16 and penile HPV-18/45.

Penile hrHPV was detected in 39 of 158 participants (24.7%) and rectal hrHPV was detected in 22 of 176 participants (12.5%); among 135 participants with testing at both sites, 9 (6.7%) had hrHPV infection at both sites (Figure 1). The most common penile hrHPV type was HPV-18/45 (n=13) followed by HPV-31/33/35/52/58 (n=10). The most common rectal hrHPV type was HPV-18/45 (n=7), followed by HPV-31/33/35/52/58 (n=6) and HPV-39–51/56/59/68 (n=5). Among the 39 participants with penile hrHPV, 89.7% (n=35) had a single detected hrHPV genotype, while 10.3% (n=4) had two detected genotype groups. Of the 22 participants with rectal hrHPV, 100% were of one type. Of 135 participants with testing at both sites, 6.7% (n=9) were co-infected with penile and rectal hrHPV.

### Factors Associated with Penile hrHPV

Penile hrHPV prevalence did not differ significantly by age, gender, or educational attainment, but was lower among employed participants (Table 1). Penile hrHPV positivity was more common among uncircumcised participants compared with circumcised participants (17.9% vs. 5.9%, *p*=0.021). HIV positive participants were more likely to have penile hrHPV than those who were HIV-negative (12.8% vs. 3.4%, *p*=0.028), though penile hrHPV did not differ by urethral STI status. Penile hrHPV prevalence was increased among men reporting insertive anal sex (p=0.122) but did not differ by any other sexual practices. In multivariable adjusted modeling (Table 3), the prevalence of penile hrHPV remained lower for participants who were employed (aPR = 0.25, p=0.048) or circumcised (aPR = 0.52, p=0.090), and increased among those who were HIV positive (aPR = 2.27, p=0.050) or practiced insertive anal sex (aPR = 5.26, p=0.062).

### Factors associated with Rectal High-Risk HPV

Rectal hrHPV prevalence did not differ by participant age, educational attainment, or employment status, but was increased among participants who were transgender or non-binary (Table 2). Rectal hrHPV was more common among HIV positive participants compared with HIV-negative participants (22.7% vs. 4.5%, *p*=0.023), and among those with rectal chlamydia and/or gonorrhea infection (22.7% vs. 1.7%, *p*=0.002). Participants reporting receptive anal sex with a male partner were significantly more likely to have rectal hrHPV than those not reporting receptive anal sex (61.7% vs. 38.3%, *p*=0.007). Condom use during receptive anal sex, number of male sexual partners in the past six months, group sex, transactional sex, lubricant use, circumcision status, and rectal hygiene practices were not significantly associated with rectal hrHPV (Table 2). In multivariable analyses (Table 3), rectal hrHPV was more likely among participants reporting receptive anal sex (aPR=3.97, *p*=0.062), having rectal chlamydia and/or gonorrhea infection (aPR=3.34, p=0.001), and being transgender/non-binary (aPR=2.28, p=0.036). HIV positivity became attenuated and non-significant due to correlation with rectal STI infection (tetrachoric rho = 0.60), but not other covariates.

## Discussion

In this cross-sectional analysis of MSM in Kenya, we observed a substantial burden of hrHPV infection at both penile and rectal anatomical sites, with distinct site-specific epidemiologic and behavioral correlates. Penile hrHPV prevalence (24.7%) was approximately twice that of rectal hrHPV (12.5%), and only a small proportion of participants with dual-site testing were co-infected. These findings add to the limited body of research in sub-Saharan Africa examining hrHPV concurrently at multiple anatomical sites among MSM and underscore the importance of site-specific epidemiologic patterns.

Our rectal hrHPV prevalence (12.5%) was lower than that reported in several other African MSM cohorts, including studies from South Africa, Mali, and the Central African Republic, where anal hrHPV prevalences ranged from approximately 48% to over 80% among MSM, and were increased among those living with HIV (Diallo et al. 2021; Nampota-Nkomba et al. 2025; Müller et al. 2016; Mboumba Bouassa et al. 2018;). However, our estimates are more comparable to findings from Benin and Rwanda, where anal or penile hrHPV prevalences ranged from approximately 20% to 36% among HIV-negative MSM (Diabaté et al. 2023; Murenzi et al. 2024). Differences in age structure, HIV prevalence, sampling strategies, and assay type, including composite genotype reporting in the ScreenFire assay, may partially explain these variations. Importantly, our cohort was relatively young and had a lower HIV prevalence (6.5%) than many previously reported African MSM cohorts, which may have contributed to the comparatively lower rectal hrHPV prevalence observed. Additionally, the majority of our cohort reported themselves to practice usually insertive sexual positioning (65%), and to also have sex with women (65.2%), which may also contribute to the higher observed prevalence of penile than anal hrHPV.

Consistent with global data (Farahmand et al. 2021; Qian et al. 2017; Zhou et al. 2023), we observed site-specific behavioral associations. Receptive anal intercourse was strongly associated with rectal hrHPV, while insertive anal sex was associated with penile hrHPV. These findings reinforce that mucosal exposure at the site of sexual contact is a key determinant of infection risk. Notably, condom use was not significantly associated with rectal hrHPV in our analysis, echoing prior studies suggesting that HPV transmission may occur despite condom use due to skin-to-skin contact beyond the area covered (Nampota-Nkomba et al. 2025; Qian et al. 2017).

HIV infection was associated with both penile and rectal hrHPV in unadjusted analyses and remained independently associated with penile hrHPV in multivariable modeling. The association between HIV and rectal hrHPV was attenuated after adjustment for rectal bacterial STI infection, reflecting strong correlation between HIV and rectal chlamydia/gonorrhea in this cohort. This finding aligns with extensive literature demonstrating synergistic interactions between HIV and HPV, where immunosuppression increases HPV acquisition and persistence, while mucosal inflammation and epithelial disruption may facilitate viral entry (NampotaNkomba et al. 2025; Müller et al. 2016; Oo, M et al., 2023; Yaya et al. 2021). Our results suggest that part of the HIV and HPV association may operate through co-occurring inflammatory STIs.

We found a strong independent association between rectal chlamydia and/or gonorrhea infection and rectal hrHPV. Participants with rectal STI had over three-fold higher prevalence of rectal hrHPV. This reaffirms the evidence that STIs disrupt epithelial integrity and may enhance HPV acquisition and persistence (Silins et al. 2005). Similar associations between laboratory-detected bacterial STIs and HPV have been reported among heterosexual men in Kenya (Smith et al. 2010) and among MSM in Europe (Ceccarani et al. 2024; Nugent et al. 2021), but data from African MSM have been limited. Our findings contribute new evidence from sub-Saharan Africa supporting integrated STI and HPV prevention approaches.

Circumcision appeared protective against penile hrHPV in adjusted analyses, consistent with prior evidence from heterosexual male cohorts in Kenya showing reduced penile HPV acquisition among circumcised men (Smith et al. 2010). A recent cohort from Nigeria hypothesized that circumcision itself may lower HPV viral load to promote penile immunity, which would increase clearance rates (Nampota-Nkomba et al. 2025). This protective association is observed in meta-analyses, demonstrating reduced incidence of HPV and increased clearance of HPV from the glans penis among circumcised men (Shapiro, et al., 2023). Among MSM, prior meta-analysis reported protective effect of circumcision against penile HPV only among HIV-infected men (Yuan, et al., 2019). Possible mechanisms by which medical male circumcision reduces risk of HPV have been previously reviewed and include increased keratinization of the glans, differences in the local immune environment and subsequent changes in the penile microbiome (Smith et al. 2010).

Interestingly, rectal hrHPV was more common among transgender and non-binary participants. This finding warrants cautious interpretation due to small subgroup numbers but may reflect structural vulnerabilities, differential access to prevention services, or distinct sexual networks.

Future research should explicitly examine HPV risk among transgender populations in East Africa, who remain underrepresented in HPV epidemiology.

We observed relatively low dual-site infection (6.7%), suggesting that penile and rectal hrHPV infections may often represent independent acquisition events rather than autoinoculation. This contrasts with some international cohorts reporting higher concordance between anatomical sites (Qian et al. 2017; Zhou et al. 2023). Differences in assay sensitivity, composite genotype grouping, and timing of infection could contribute to these discrepancies. Differences in behavioral practices may also be a factor, as only 19.2% of participants in our cohort being usually versatile in sexual positioning.

Our findings highlight several important implications for HPV prevention among MSM in Kenya and similar LMIC settings. First, the burden of hrHPV in young MSM, many of whom are HIV-negative, supports expansion of gender-neutral HPV vaccination policies that include MSM before or soon after sexual debut (Lorway et al., 2022). The frequent detection of genotypes beyond HPV-16/18 in African cohorts (Mboumba Bouassa et al. 2018; Diabaté et al. 2023) further underscores the value of nonavalent vaccination where feasible. Second, the strong association between rectal bacterial STIs and rectal hrHPV supports integration of etiologic STI screening (rather than syndromic management alone) with HPV prevention services. Given high rates of asymptomatic chlamydia and gonorrhea infection among Kenyan MSM (Otieno et al. 2020; Mofolorunsho et al. 2024), routine rectal screening may represent a key opportunity for reducing both STI-related morbidity and HPV-associated cancer risk. Finally, our results reinforce the need for anal cancer prevention strategies in high-risk MSM populations in LMICs, including consideration of anal cytology or high-risk HPV–based screening in settings where resources permit. While cervical cancer elimination efforts have accelerated globally (WHO 2024), anal cancer prevention remains largely neglected in sub-Saharan Africa.

## Strengths and Limitations

This study has several strengths. We measured penile and rectal hrHPV using molecular testing, concurrently assessed laboratory-confirmed bacterial STIs, and collected detailed behavioral data. The use of validated onsite testing platforms demonstrates feasibility of integrated HPV and STI diagnostics in Kenyan clinical settings. Limitations should be acknowledged. First, the cross-sectional design cannot distinguish incident from persistent infection. Second, the AmpFire assay reports some genotypes in composite groups, limiting precise type-specific analysis and there may have been multiple genotype infections that were masked by this. Third, a proportion of early samples were invalid, reducing sample size and representation of the sample, though invalidity decreased as laboratory expertise improved. Fourth, HIV prevalence in our cohort was relatively low compared to other MSM cohorts in the region, which may limit generalizability to higher-HIV-prevalence populations. Finally, behavioral practices were self-reported and may be subject to social desirability bias.

## Conclusions

In this study, hrHPV infection was common among MSM in Kenya, with distinct behavioral and biological correlates by anatomical site. The strong association between rectal bacterial STIs and rectal hrHPV, and the association between HIV and penile hrHPV, highlight the importance of comprehensive, integrated sexual health services. Strengthening programs that combine etiologic STI testing, HIV care, and HPV prevention, including expansion of gender-neutral HPV vaccination, may be critical to reducing HPV transmission and subsequent HPV-associated cancer risk among MSM in sub-Saharan Africa.

## Supplementary Material

Table 1 to 3 are available in the Supplementary Files section.

Supplementary Files

This is a list of supplementary files associated with this preprint. Click to download.

- FinalMbiliPamojaHRHPVTables13.docx
- FinalSupplementalTable1.docx

## Data Availability

The datasets used and/or analyzed during the current study are available from the corresponding author on reasonable request.

## List of Abbreviations

DNA: Deoxyribonucleic acid
HIV: Human Immunodeficiency Virus
hrHPV: High Risk Human papilloma Virus
MSM: Men who have Sex with Men
STI: Sexually transmitted infection
WHO: World Health Organization
